# Immune Thrombocytopenic Purpura and Intracranial Stenting

**DOI:** 10.1177/08971900241236121

**Published:** 2024-02-22

**Authors:** Sophia Pathan

**Affiliations:** 1 Department of Pharmacy, 21798 Hospital of the University of Pennsylvania, Philadelphia, PA, USA

**Keywords:** immune thrombocytopenic purpura, intracranial stents, antiplatelet therapy, dual antiplatelet therapy, stents

## Abstract

Patients with immune thrombocytopenic purpura (ITP) presenting with indications for dual antiplatelet therapy (DAPT) can be difficult to manage due to the precarious balance of managing the need for increased platelet counts as well as inhibition of platelet activity. This case represents a 65 year old woman with ITP who presented with a bilateral subarachnoid hemorrhage secondary to a left ophthalmic aneurysm that required placement of a pipeline embolization device (PED) necessitating DAPT. After treatment of her ITP with pulse dexamethasone for four days, she was safely discharged on one month of DAPT with aspirin and ticagrelor then switched to aspirin monotherapy without any immediate complications. During her period of DAPT, she did not receive additional medical treatment for her ITP. This case successfully presents a high-risk ITP patient requiring DAPT for a neurosurgical procedure and illustrates that these patients can be safely and successfully treated with DAPT once their ITP is stabilized.

## Introduction

Thrombocytopenia is a deficiency in platelet count classified as mild (100 to 150 × 10^9^/L), moderate (50 to 100 × 10^9^/L), or severe (< 50 × 10^9^/L).^1-3^ Thrombocytopenia can have many etiologies, but one of the more common causes is idiopathic or immune thrombocytopenic purpura (ITP).^1^ ITP is a platelet disorder characterized by autoantibody-mediated destruction of platelets and can result in thrombocytopenia and bleeding complications, such as purpura and petechiae.^1,2^ Patients with ITP need to be monitored for their platelet counts, and risks for bleeding in patients with indications for antiplatelet therapy should be monitored in particular.

In cardiac patients, dual antiplatelet therapy (DAPT) in acute coronary syndrome (ACS) evaluates platelet count, platelet function, and whether the thrombocytopenia is secondary to decreased platelet production, increased platelet consumption, or another etiology such as platelet sequestration.^4^ There is currently no guideline on management of DAPT in thrombocytopenic patients, particularly in ITP, though there are consensus recommendations from the European Society of Cardiology (ESC).^5^ ESC recommendations include that stented patients with ACS who do not have severe thrombocytopenia should receive DAPT with aspirin and clopidogrel for one month followed by a single antiplatelet agent afterward.^5^ Clopidogrel monotherapy is recommended over aspirin monotherapy due to a statistically significant relative risk reduction in gastrointestinal bleeding with clopidogrel as opposed to aspirin 325 mg monotherapy.^5,6^ Prasugrel or ticagrelor is not usually recommended in thrombocytopenic DAPT regimens due to increased bleeding risk, though there is little data on use as monotherapy or in non-cardiac indications.^5,7,8^

In patients with intracranial aneurysms, treatment can involve the use of a pipeline embolization device (PED).^9,10^ The PED is a microcatheter-delivered braided wire mesh cylinder that is placed within the internal carotid artery in the brain to block off large or wide-necked aneurysms.^9,10^ The endovascular mesh of the PED acts as a scaffold for endothelial overgrowth and forms a layer that covers the aneurysm neck over time.^9^ As with cardiac stents, patients with PEDs require DAPT regimens post-procedure.^9^ Pipeline thrombosis can be associated with catastrophic morbidity and mortality.^11^

Imbalance in the treatment of increasing platelet counts by inhibiting the activity of autoantibodies while simultaneously inhibiting platelet activity with P2Y12 antagonists can result in life-threatening complications. Current case reports detail patients with ITP and DAPT for ACS using clopidogrel or ticagrelor.^12,13^ There is limited data on shorter durations of DAPT in cerebrovascular patients with PEDs, with case reports detailing the possibility but no randomized controlled trials providing true guidance.^14^ This case represents a patient with ITP and a need for DAPT utilizing ticagrelor after neurosurgery.

## Case

A 65 year old woman presented with a history of hypertension, remote thyroid cancer, Sjögren’s syndrome (since 2012, symptoms including sicca, parotid fullness, and possible arthritis), and ITP with autoantibody profile ANA, SSA+, and low C4.

Her home medications did not significantly impact platelets. For her Sjögren’s syndrome, her medications included prednisone 5 mg daily (for which she was tapering to be off shortly), bimatoprost 0.01% eye drops with one drop in both eyes every evening, brimonidine 0.1% eye drops with one drop in both eyes every evening, cyclosporine 0.05% eye drops with one drop in both eyes twice daily, and dorzolamide-timolol 22.3-6.8 g eye drops with one drop in both eyes every morning. She managed her pain with duloxetine 30 mg every bedtime, gabapentin 300 mg every bedtime, and meloxicam 7.5 mg daily. For her hypertension, she was taking amlodipine 10 mg daily, chlorthalidone 25 mg daily, and losartan 100 mg daily. For her ITP, she was followed closely by hematology outpatient and completed three doses of rituximab 6 weeks prior to her hospitalization with good response. She had also received intravenous immune globulin (IVIG) as an outpatient several times but none within the month prior to admission.

She presented to an outside hospital with headache and thrombocytopenia with a nadir of 8 × 10^9^/L. At the outside hospital, she received several platelet transfusions, broad spectrum antibiotics due to concern for meningitis, aminocaproic acid, and IVIG. She acutely worsened, however, and the computed tomography (CT) scan of her head showed bilateral subarachnoid hemorrhage and subdural hemorrhage, so she was transferred to the neurosciences intensive care unit (ICU) at our institution for further management.

**Figure 1.**
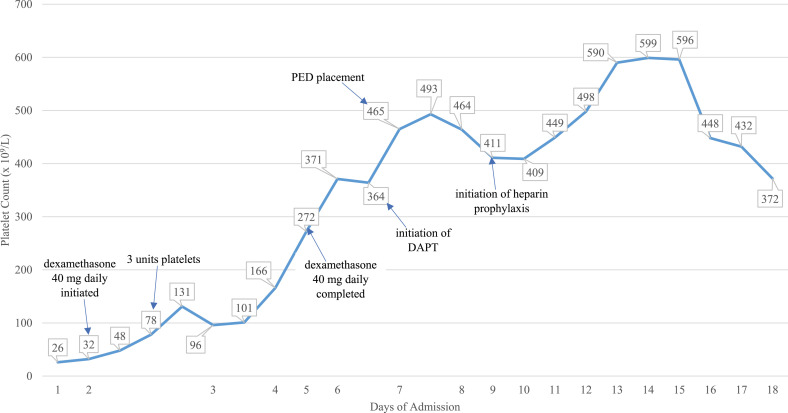
Platelet trend during admission.

On arrival, her platelet counts were 26 × 10^9^/L and she received an additional three units of platelets. Hematology was consulted for her ITP management, and she was started on pulse dexamethasone 40 mg daily for 4 days. Afterward, platelet transfusions were reserved for bleeding episodes only, with the expectation that her platelet count would not durably rise secondary to transfusion. Figure 1 details her platelet trends through admission.

On her second day of admission, she underwent a digital subtraction angiography scan and was found to have a left ophthalmic internal carotid aneurysm and mild bilateral anterior circulation vasospasm. The decision was made to manage her as though she had a ruptured aneurysm, and plans were underway to place a PED near the aneurysm. For this neurosurgical procedure, however, her platelets would need to be stabilized, and the decision of a DAPT regimen and duration needed to be made. Since she followed closely with her outpatient hematologist, there was discussion between inpatient and outpatient hematology. Initially, inpatient hematology recommended against DAPT due to her severe ITP, but neurosurgery did not feel comfortable putting in a PED without a plan for DAPT post-procedure. As her platelet counts improved from the pulse steroid regimen with dexamethasone, inpatient and outpatient hematology collaborated and determined it would be safe and efficacious to treat her with DAPT for 30 days followed by aspirin monotherapy for at least 6 months.

Her home medications for her pain and Sjögren’s syndrome were all continued inpatient, but her home prednisone was held due to the initial concern for infection and her initial 4 days of intravenous dexamethasone 40 mg daily. She was not restarted on prednisone after the dexamethasone pulse regimen due to her bleed. Additionally, her home blood pressure medications were held in the peri-operative period.

On day six of admission, she went to the operating room for placement of a PED for her aneurysm. She was loaded with ticagrelor 180 mg on the night prior to the procedure and received no load for her aspirin. Afterward, she was started on aspirin 81 mg daily and ticagrelor 90 mg twice daily as her DAPT regimen starting on day seven. Using a VerifyNow P2Y12 device, her platelet reactivity units were measured at 42 and 50, showing adequate platelet inhibition with ticagrelor.

Post-procedure, there was discussion about the safety of restarting venous thromboembolism prophylaxis as her platelet counts had stabilized and DAPT does not provide coverage for thromboembolism. Her activated partial thromboplastin time (aPTT) values had been prolonged through her admission, with a peak of 52.5 s that did not correct with a mixing study. Additionally, her factor VIII was 213, factor IX was 120, factor XI was 90, and factor XII was 63, results that were consistent with a factor inhibitor. As inhibitors can develop in patients with autoimmune disorders and acute illness and her intrinsic pathway factor activity levels were all normal, the inhibitor was likely not directed against a coagulation factor and was probably akin to an anti-phospholipid antibody. Anti-phospholipid antibodies can prolong the PTT *in vitro* but may not have an *in vivo* effect. Thus, with hematology approval, she was placed on DAPT with subcutaneous heparin as prophylaxis. Rather than full dose subcutaneous heparin, though she was started on a lower dose of 2500 units every 8 hours. She also experienced some post-procedure thrombocytosis that ultimately stabilized.

There was discussion about restarting her home prednisone for her Sjögren’s syndrome, as it would provide immunosuppression for her ITP, but due to her presenting intracranial bleed and her stable platelet counts, continuation of her home prednisone was deferred. Additionally, she was meant to be tapered off the regimen outpatient, so there was no need to restart it just for the ITP. She was thus on DAPT and venous thromboembolism prophylaxis with no further immunosuppression for ITP.

Three days after placement of her PED, on hospital day 10, she was transferred to the step down unit and optimized for discharge. She was discharged after 18 days of admission to a rehabilitation facility on aspirin 81 mg daily and ticagrelor 90 mg every 12 hours. After the patient had been on DAPT with aspirin and ticagrelor for 30 days, she was switched to aspirin 81 mg daily as monotherapy. At her weekly follow-up visits in her hematology clinic, her platelets remained within normal limits, and she was not restarted on immunosuppression. She continues to deny bleeding events and has continued on aspirin monotherapy at this time.

## Discussion

In ITP patients, there is no consensus on the safest platelet count to maintain prior to DAPT, the ideal combination, and the optimal duration of antiplatelet agents. Shah AH et al. wrote of a patient with a platelet nadir of 35 × 10^9^/L that improved to 85 × 10^9^/L with IVIG, which was when DAPT was initiated and the patient went for percutaneous coronary intervention.^13^ Shah MU et al. described a patient who received prednisolone 95 mg daily to improve platelet counts to 102 × 10^9^/L prior to angiography.^12^ Consensus leans toward maintaining platelets at least at moderate thrombocytopenia prior to procedures. Following this recommendation, our patient did not go for her procedure until she was no longer thrombocytopenic at a platelet count of 493 × 10^9^/L, which was done with outpatient rituximab, IVIG, pulse dexamethasone, and platelet transfusions.

Although the cause of neurologic affectation in this patient was the aneurysm, it is also important to consider the possibility of a complication of refractory ITP. In this case, the patient continued to worsen after the initial IVIG, had a history of recurrent IVIG and rituximab use, and was already on steroid maintenance therapy. Ruling out an ITP complication has the potential of saving resources so is important to consider on patient presentation as a differential diagnosis.

Though most data in ITP patients requiring DAPT is in ACS patients, management of DAPT can potentially be extrapolated to the cerebrovascular population due to similarities in patient acuity and stent management. Data recommend monotherapy with clopidogrel after at least one month of DAPT. In the case report by Shah AH et al, their patient was discharged with a plan for aspirin and clopidogrel as DAPT and aspirin as long-term monotherapy, but as the patient experienced stent thrombosis within 24 hours of discharge on this regimen, the patient was maintained on DAPT with aspirin and ticagrelor for 12 months.^13^ Shah MU et al. presented a case where a thrombocytopenic patient had a non-ST segment elevation myocardial infarction and ITP and was managed initially with clopidogrel monotherapy then loaded with aspirin and transitioned to DAPT when the platelet numbers were greater than 50 × 10^9^/L. The patient had DAPT for one month followed by clopidogrel monotherapy.^12^ Our patient had DAPT therapy for one month post-PED, but we utilized aspirin monotherapy, which does not follow ACS management protocols. Thus far, our patient has not experienced any bleeding or thrombotic adverse effects. Our patient could have been managed with aspirin and clopidogrel as DAPT followed by clopidogrel monotherapy, but general practice for cerebrovascular patients utilizes aspirin monotherapy.^15,16^

As ITP is an autoantibody process, patients are managed with immunosuppression to increase platelet counts. In the report by Shah AH et al, their patient was maintained on prednisolone, azathioprine, and romiplostim with DAPT to concomitantly manage ITP, and their patient had a history of clotting secondary to IVIG.^13^ Though IVIG can work more rapidly than glucocorticoids for ITP, efficacy is considered similar between the two agents.^1,2^ A major concern with IVIG, however, is the risk of thrombosis, thought to be secondary to its viscosity or its platelet agonist properties.^17,18^ Paradoxically, ITP has also been associated with thrombosis and sufficient prophylaxis measures should be taken to address possible thromboembolic effects whenever possible.^19^ Therefore, the risk of thrombosis from ITP treatment needs to be balanced with the risk of thrombosis from antiplatelet monotherapy rather than DAPT in managing cerebrovascular stents. Our patient had received IVIG, but she needed additional treatment with glucocorticoids. After the dexamethasone, there was discussion on whether to restart her glucocorticoids. Since other reports had continued ITP treatment while patients were on DAPT, our patient may have experienced benefit from further ITP treatment. She could theoretically have received a longer duration of DAPT if maintained on immunosuppression. Due to her brain bleed, however, there was concern for continuing glucocorticoids, so the risks and benefits were weighed and she did not continue on immunosuppressive agents, shortening DAPT duration.

In cardiac stenting procedures, there are a variety of stents available. Previous practice included use of bare metal stents to allow high bleeding risk patients to utilize a shorter duration of DAPT.^20,21^ More recent trials have shown that second generation drug-eluting stents have better outcomes, with third generation drug-eluting stents being the preferred choice in high bleeding risk patients.^22,23^ These approaches can allow for a shorter duration of DAPT prior to monotherapy, and later generation drug-eluting stent usage was the method that Shah MU and Shah AH et al undertook to minimize DAPT duration and optimize outcomes in their patients. In cerebrovascular procedures, choice of device is often dependent on the type of aneurysm, meaning that the duration of DAPT would need to be optimized rather than the device or procedure. Optimal duration of DAPT post-PED is therefore unclear.^15^ Surveys of neurointerventionalists at different institutions have revealed varying regimens for DAPT duration, from one month to one year of DAPT prior to aspirin monotherapy, with clopidogrel as the most common P2Y12 agent.^15,16^ For our patient, due to her risk factors, DAPT duration was shortened to one month; however, as she was high risk for thrombosis, she was started on ticagrelor.

Post-PED, many centers will use VerifyNow platelet function testing to determine P2Y12 inhibitor effect for potential DAPT adjustment.^15,16^ About one-third of patients may have clopidogrel resistance, which may result in an increased risk of thromboembolic complications.^16,24^ Utilizing a VerifyNow P2Y12 device to measure P2Y12 activity for our patient’s ticagrelor maintenance doses, our patient’s platelet reactivity units were measured at 42 and 50 (reference range for efficacy, less than 194), showing adequate platelet inhibition.

This case represents the rare but difficult scenario of managing ITP patients presenting with an indication for DAPT and is one of the first to report a patient requiring DAPT for a cerebrovascular indication. Managing ITP patients with cerebrovascular indications for DAPT remains challenging and underreported. Initial management should include treatment of ITP until patients are no longer severely thrombocytopenic, utilizing medications such as glucocorticoids, IVIG, and other immunosuppressants. Afterward, there should be discussion with a hematology specialist to determine the ideal agents for DAPT for the specific clinical scenario, as well as the safest duration for both bleeding and thrombotic risks. Further reports and studies should be done to guide future management of DAPT in ITP patients.

## Conclusion

Antiplatelet drugs are necessary in management of stent thrombosis, but their benefits need to be weighed with the risks of bleeding, particularly in patients with ITP. Patients with ITP and a cerebrovascular indication for DAPT, such as placement of a PED, should be monitored closely but can potentially tolerate DAPT utilizing ticagrelor.
